# Recurrent Anterior Chamber Migration of Intravitreal Triamcinolone Following Scleral‐Fixated IOL Implantation

**DOI:** 10.1002/ccr3.71102

**Published:** 2025-10-31

**Authors:** Yuki Takagi, Sho Yokoyama, Kazunori Takeuchi

**Affiliations:** ^1^ Department of Ophthalmology Japan Community Healthcare Organization Chukyo Hospital Nagoya City Aichi Prefecture Japan

**Keywords:** intraocular lens, intraocular pressure, scleral fixation, triamcinolone acetonide

## Abstract

In eyes with a scleral‐fixated IOL, intravitreal triamcinolone may migrate into the anterior chamber, resulting in early‐onset elevation of IOP, even at small volumes. Prompt slit lamp and gonioscopic evaluation is recommended for patients with prior vitrectomy or posterior capsule defects to ensure timely detection and treatment.

## 1 Introduction

Triamcinolone acetonide is widely used as a vitreous dye to visualize the vitreous body during vitrectomy procedures [1, 2], as well as for the treatment of macular edema associated with diabetic retinopathy and retinal vein occlusion [3, 4]. Known complications following its administration include intraocular pressure (IOP) elevation and endophthalmitis. In particular, IOP elevation has been reported in approximately 50% of cases [5, 6], typically occurring approximately 1–2 months after triamcinolone administration [3, 4, 6]. In most cases, the elevation is transient and can be controlled with topical medications, with improvement generally seen within 6 months. However, some cases may require filtration surgery [5], and in rare instances, serious visual impairment may persist [7].

Although rare, anterior chamber migration of intravitreally injected triamcinolone has been reported [8, 9]. In some cases, a large volume of triamcinolone migrating into the anterior chamber has been reported to cause angle closure and subsequent IOP elevation [9]. However, to our knowledge, there have been no reports of repeated anterior chamber migration, nor of IOP elevation associated with a small amount of migration. Here, we report a case in which a small volume of triamcinolone repeatedly migrated into the anterior chamber, resulting in early‐onset IOP elevation following administration.

## 2 Case History and Examination

A 52‐year‐old man with a history of type 1 diabetes began treatment for diabetic macular edema in November 2017. Between 2018 and March 2023, the right eye received two intravitreal injections of ranibizumab and six intravitreal injections of triamcinolone acetonide (IVT) (4 mg/0.1 mL), as well as one sub‐Tenon triamcinolone acetonide (STTA) injection. The left eye received one intravitreal injection of brolucizumab, 14 IVT injections, and one STTA injection.

In June 2019, during follow‐up, the patient underwent bilateral cataract surgery. In the left eye, a posterior capsule rupture was observed, necessitating anterior vitrectomy. Although the intraocular lens (IOL) was initially fixed to the ciliary sulcus, IOL displacement was noted, prompting scleral fixation and vitrectomy a month later.

Following IVT in the left eye—specifically after the first and second injections (prior to cataract surgery), and the third and fifth injections (post‐scleral IOL fixation)—elevated IOP (IOP > 21 mmHg) was observed approximately 1 month after injection, despite the absence of overt anterior chamber inflammation or visible foreign bodies. The elevated IOP was managed with topical anti‐glaucoma medications and oral acetazolamide, resulting in an improvement in the condition. In August 2022, the 14th IVT was administered to the left eye. Subsequently, the IOP was managed with tafluprost monotherapy.

In March 2023, the recurrence of macular edema in the left eye prompted the 15th IVT. Prior to the injection, best‐corrected visual acuity was 20/12.5 in the right eye and 20/125 in the left eye. IOP, measured by noncontact tonometry (NCT), was 15 mmHg in the right eye and 18 mmHg in the left eye.

Two days after the injection, the patient noticed whitening of the black part of the eye and presented for evaluation 1 week postinjection. At the time of examination, he reported no photophobia or ocular pain, but IOP by NCT was elevated in the left eye (18 mmHg in the right eye and 44 mmHg in the left eye).

**FIGURE 1:**
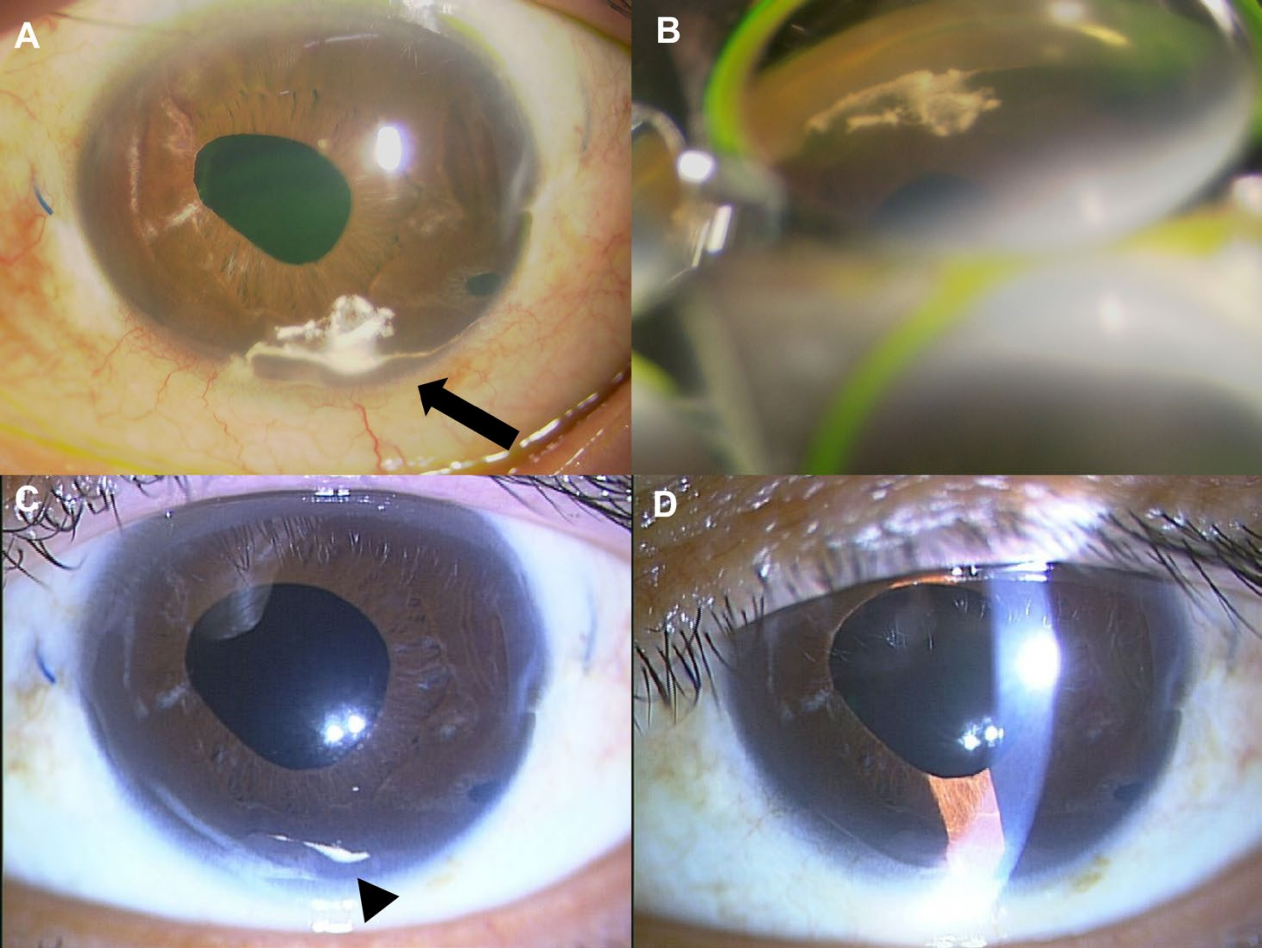
Clinical course following the first anterior chamber migration of triamcinolone. (A) Slit lamp photograph at 1 week postinjection showing triamcinolone accumulation around preexisting peripheral anterior synechiae (PAS) in the inferior anterior chamber (arrow). (B) Gonioscopic image of the inferior quadrant showing PAS with surrounding triamcinolone deposits. (C) Slit lamp photograph at 3 weeks postinjection showing slight residual triamcinolone (arrowhead). (D) Slit lamp photograph at 2 months postinjection showing complete resolution of triamcinolone.

Slit lamp examination revealed numerous white particles in the anterior chamber, presumed to be triamcinolone (Figure 1). Gonioscopy revealed previously known peripheral anterior synechiae and newly visible triamcinolone granules in the inferior angle, with no abnormalities in other quadrants.

## 3 Differential Diagnosis, Investigations, and Treatment

Based on these findings, the IOP elevation was attributed to triamcinolone migration. Anterior chamber paracentesis and oral acetazolamide (250 mg × 2 tablets) were administered. Two weeks later, triamcinolone remained visible in the anterior chamber, but IOP had decreased to 13 mmHg.

## 4 Outcome and Follow‐Up

Two months after the March 2023 injection, triamcinolone had completely disappeared from the anterior chamber, and IOP remained stable at approximately 20 mmHg without further treatment.

**FIGURE 2:**
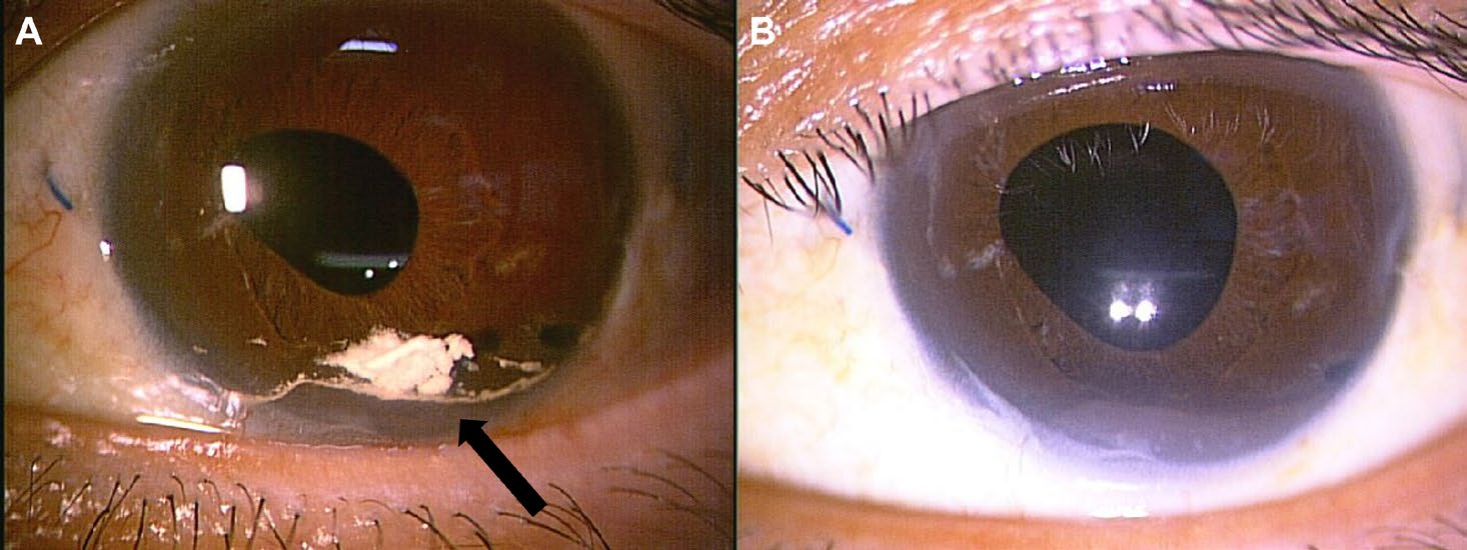
Clinical course following the second anterior chamber migration of triamcinolone. (A) Slit lamp photograph at 2 weeks postinjection showing triamcinolone accumulation in the inferior anterior chamber (arrow). (B) Slit lamp photograph at 2 months postinjection showing slight residual triamcinolone.

**FIGURE 3:**
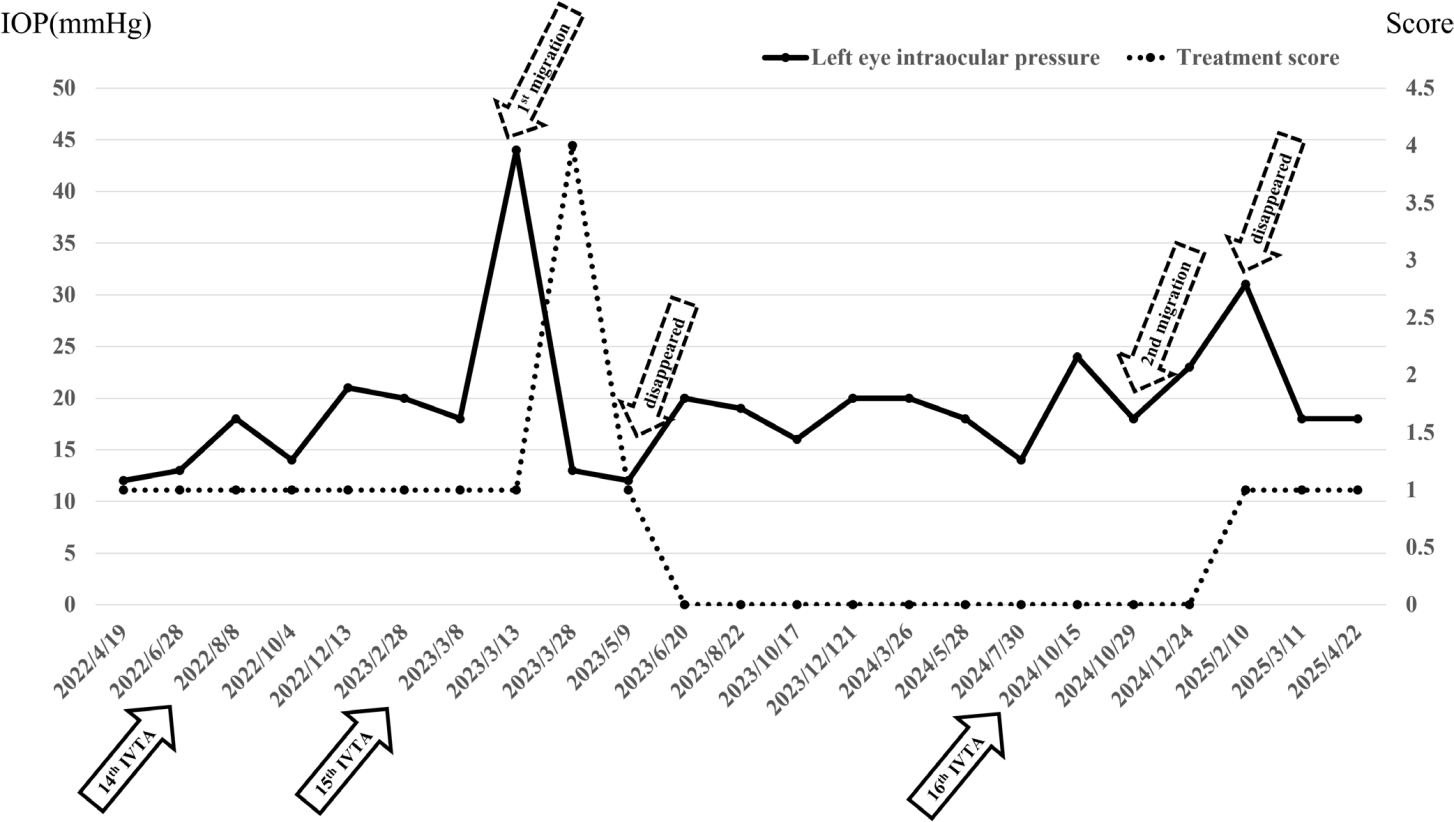
Timeline of clinical events following the 14th intravitreal triamcinolone injection. Timeline depicting the intravitreal triamcinolone injections after the 14th dose (solid arrows), changes in intraocular pressure (solid line), and treatment score (dotted line: 1 point per single‐agent eye drop, 2 points per combination drop, 1 point per acetazolamide tablet). Dotted arrows indicate the timing of triamcinolone appearance and disappearance in the anterior chamber.

In October 2024, macular edema recurred in the left eye, and IVT was repeated. At the 2‐week follow‐up, IOP was 18 mmHg, but anterior chamber migration of triamcinolone was observed again (Figure 2). At 2 months, IOP was slightly elevated at 22 mmHg, with no signs of inflammation, and triamcinolone was still present. By 4 months postinjection, triamcinolone had disappeared, but IOP had increased to 30 mmHg. Tafluprost eye drops were initiated, resulting in rapid IOP reduction. The IOP trend and treatment course, including eye drop therapy, are shown in Figure 3.

## 5 Discussion

In the present case, we observed repeated migration of triamcinolone into the anterior chamber following IVT. While known complications of IVT include sterile endophthalmitis with hypopyon formation and IOP elevation, reports of triamcinolone migration into the anterior chamber accompanied by IOP elevation are rare. To the best of our knowledge, no previous reports have documented such migration occurring on multiple occasions. A summary of previously reported cases, compared with the present case, is shown in Table 1.

**TABLE 1:** Summary of reported cases of anterior chamber migration of intravitreal triamcinolone and comparison with the present case.

Case	Age sex	Lens status	Vitrectomy history	Triamcinolone dose	Time to migration into anterior chamber	Volume of triamcinolone in anterior chamber	Timing of intraocular pressure elevation
Ruiz‐Moreno et al. [8]	78 Female	Phakic	None	20 mg	1 day	Extending to the pupil	None
Tan et al. [9]	52 Female	Sulcus lens implant	Yes (anterior vitrectomy for posterior capsule rupture)	15 mg	1 week	Covering approximately 120 degrees of angle (inferior to temporal quadrant)	1 week
Present case: First migration episode	52 Male	Scleral‐fixated intraocular lens	Yes	4 mg	1 week (subjective symptoms began on day 2)	Small amount in inferior quadrant	1 week
Present case: Second migration episode	52 Male	Scleral‐fixated intraocular lens	Yes	4 mg	2 weeks	Small amount in inferior quadrant	4 months

Steroid‐induced IOP elevation is thought to involve increased aqueous outflow resistance due to remodeling of the trabecular meshwork [10]. IOP elevation following triamcinolone administration typically occurs approximately 1–2 months postinjection [3, 4]. Considering that remodeling of the trabecular meshwork requires time to develop, delayed IOP elevation is generally attributed to this mechanism.

However, in the present case—as well as in a previously reported case involving anterior chamber migration of triamcinolone [9]—IOP elevation was observed approximately 1 week after injection, earlier than the typical onset associated with steroid‐induced trabecular meshwork remodeling. This suggests that alternative mechanisms may be involved. In a previous report [9], triamcinolone was noted to occupy more than 120° of the anterior chamber angle, suggesting that secondary angle closure may have contributed to the IOP elevation.

Although angle closure may also have contributed to IOP elevation in our case, there are notable differences. The previously reported case involved a 15 mg dose of triamcinolone, whereas our patient received only 4 mg. Additionally, a small volume of residual triamcinolone was observed in our case compared to that of the slit lamp images presented in the prior report. These differences suggest that other mechanisms may be involved.

Given the small amount of triamcinolone observed in the anterior chamber, it is possible that fine triamcinolone particles mechanically obstructed the trabecular meshwork, thereby reducing aqueous outflow and leading to IOP elevation. This implies a secondary open‐angle glaucoma–like mechanism.

However, no IOP elevation was observed during the second episode of triamcinolone migration. A key difference from the first episode is that no migration into the anterior chamber was noted at 1 week postinjection, whereas migration was detected at 2 weeks postinjection.

Although the patient had previously undergone vitrectomy, some residual vitreous likely remained in the peripheral region. It is speculated that, during the first episode, finely dispersed triamcinolone particles that were not entangled in the residual vitreous migrated into the anterior chamber early. By contrast, during the second episode, the triamcinolone may have clumped together and become partially entrapped in the residual vitreous, delaying its migration into the anterior chamber.

When fine, dispersed triamcinolone particles enter the anterior chamber, they are more likely to obstruct the pores of the trabecular meshwork, impairing aqueous outflow and resulting in IOP elevation. By contrast, if triamcinolone migrates in a more aggregated form after a delay, it may block aqueous outflow at the site of migration without affecting outflow in other quadrants, thereby avoiding immediate IOP elevation. In such cases, subsequent IOP elevation may occur later due to delayed trabecular meshwork remodeling. These clinical findings suggest that the mechanism of IOP elevation may vary depending on the timing of its onset.

Animal models have demonstrated that injecting microbeads into the anterior chamber of mice elevates IOP by physically blocking the trabecular outflow pathway [11]. However, whether triamcinolone can induce a similar effect has yet to be reported. Further experimental studies to investigate this possibility are needed.

In this case, the patient had undergone vitrectomy, which may have facilitated the migration of triamcinolone into the anterior chamber. A previous report has documented vitreous prolapse into the anterior chamber following IVT, even after vitrectomy, suggesting that substances within the vitreous cavity may readily migrate anteriorly in eyes with posterior capsule rupture or prior vitrectomy [12]. Therefore, in such eyes, clinicians should consider the possibility of anterior chamber migration of triamcinolone following IVT. Early postoperative examination is warranted to assess intraocular pressure elevation.

In conclusion, in eyes with scleral‐fixated IOL, IVT may migrate into the anterior chamber. When such migration occurs shortly after injection, it may lead to early‐onset IOP elevation, highlighting the need for close monitoring and prompt intervention.

## Author Contributions

**Yuki Takagi:** conceptualization, investigation, visualization, writing – original draft. **Sho Yokoyama:** supervision, visualization, writing – review and editing. **Kazunori Takeuchi:** writing – review and editing.

## Consent

The patient provided written informed consent for the publication of her examination and imaging findings for educational, research, and quality improvement purposes.

## Conflicts of Interest

The authors declare no conflicts of interest.

## Data Availability

The authors have nothing to report.
